# Prediction of gastrointestinal symptoms trajectories using omega-3 and inflammatory biomarkers in early-stage breast cancer patients receiving chemotherapy

**DOI:** 10.1007/s00520-023-08274-5

**Published:** 2024-01-03

**Authors:** Daniela Arcos, Ding Quan Ng, Yu Ke, Yi Long Toh, Alexandre Chan

**Affiliations:** 1 School of Pharmacy & Pharmaceutical Sciences, University of California Irvine, Irvine, USA; 2 Division of Supportive and Palliative Care, National Cancer Centre Singapore, Singapore, Singapore; 3 Department of Pharmacy, National University of Singapore, Singapore, Singapore

**Keywords:** Inflammation, Omega-3, Gastrointestinal symptoms, Breast cancer, Chemotherapy

## Abstract

**Purpose:**

Gastrointestinal (GI) symptoms are common among breast cancer patients undergoing chemotherapy, negatively impacting treatment outcomes and quality of life. Evidence points to inflammatory processes as the underlying cause of chemotherapy-associated GI symptoms. Relatedly, omega-3 (n-3) has been linked to anti-inflammatory processes. The primary objective of this study was to examine the associations between baseline n-3, baseline inflammatory markers and GI symptom progression in early-stage breast cancer patients receiving chemotherapy.

**Methods:**

In this secondary analysis of a prospective cohort study, we analyzed baseline levels of inflammatory biomarkers (measured using a Luminex bead-immunoassay) and plasma levels of DHA, EPA, and FFA (measured using enzyme-linked immunosorbent assay). GI symptoms were assessed using the European Organisation for Research and Treatment of Cancer Quality of Life Questionnaire in Cancer Patients (EORTC QLQ-C30) symptom scale scores at baseline (T1) and at least 6 weeks after, during chemotherapy (T2). Inferential statistics were used to analyze associations between the variables of interest.

**Results:**

The analysis included 31 female breast cancer patients (mean age ± SD = 50.5 ± 8.8; 89.6% receiving anthracycline-based chemotherapy). Higher levels of docosahexaenoic acid (DHA) and interleukin-8 (IL-8) predicted increases in appetite loss. Similarly, higher IL-8 predicted worsened nausea and vomiting.

**Conclusion:**

Baseline IL-8 and DHA predicted GI symptom progression in early-stage breast cancer patients undergoing chemotherapy. Future studies are required to evaluate how therapeutic intervention targeting these biomarkers may mitigate gastrointestinal symptoms in cancer patients.

**Supplementary Information:**

The online version contains supplementary material available at 10.1007/s00520-023-08274-5.

## Introduction

Chemotherapy plays an important role in the treatment of early-stage breast cancer. However, its side effects can negatively impact the quality of life (QoL) during and after treatment [1, 2]. Among these side effects, gastrointestinal (GI) symptoms, such as decreased appetite, nausea and vomiting, abdominal pain, bloating, diarrhea, and constipation, are frequently reported among breast cancer patients undergoing anticancer treatment [3–5]. GI symptoms, besides being the most common side effects of cancer treatment, can have a detrimental impact in patients’ functioning and QoL [6, 7]. These symptoms can significantly impair the patient’s ability to carry out daily activities and engage in social interactions and have been associated with increased rates of psychological distress [6–8]. Addressing and properly managing GI symptoms improve QoL and overall well-being, proving to be an important aspect of cancer care.

Inflammation has been identified as a key factor contributing to GI symptoms in cancer patients [9–12]. Chemotherapy agents, while effective in destroying cancer cells, can lead to intestinal inflammation. By destroying gut microbes and irritating intestinal lining, which increases the permeability of the intestines, chemotherapeutic agents lead to the activation of immune cells and the release of pro-inflammatory cytokines [13]. Relatedly, the imbalance in the gut microbiome induced by chemotherapy can lead to an increased production of pro-inflammatory cytokines and a decrease in anti-inflammatory cytokines, exacerbating inflammation and leading to the development of GI toxicities [14]. Simultaneously, tumor cells trigger the release of pro-inflammatory mediators when destroying healthy cells, leading to inflammation [15]. In other words, in patients undergoing cancer treatment, inflammation can be caused by both the disease and the therapy which, in turn, leads to a wide array of symptoms, including GI toxicities.

Strategies to reduce inflammation may be beneficial in mitigating GI toxicities. One potential approach is the supplementation of omega-3 (n-3) polyunsaturated fatty acids (PUFAs), which have been shown to have anti-inflammatory properties. Eicosapentaenoic acid (EPA) and docosahexaenoic acid (DHA), two n-3 PUFAs commonly found in oily fish, have been observed to lower inflammation through various mechanisms, including inhibiting production of pro-inflammatory cytokines and activating inflammation-resolving mediators [16]. Importantly, clinical studies have shown that dietary n-3 PUFAs increase n-3 fatty acid levels in red blood cells, plasma, and leukocyte membranes and shift eicosanoid synthesis towards less pro-inflammatory species [17, 18]. In a meta-analysis of 49 published clinical trials regarding n-3 PUFAs and cancer, it was observed that n-3 PUFAs have a positive effect on inflammation and cachexia, possibly by regulating inflammatory response, neuron cell survival, and skeletal muscle protein turnover [19]. Considering this, it is expected that increased levels of n-3 PUFAs would be associated with reduced inflammation and, therefore, would also lead to lower risk of GI symptomatology.

It is important to gain further insights on the relationship between n-3 PUFAs, inflammation, and GI symptomatology in cancer patients. Specifically for early-stage breast cancer patients undergoing chemotherapy, it is currently unclear whether inflammation and n-3 PUFAs biomarkers contribute to the progression of GI symptoms. Hence, this study aims to investigate the relationship between baseline levels of pro-inflammatory cytokines and n-3 PUFAs, and the progression of GI symptoms in early-stage breast cancer patients undergoing adjuvant chemotherapy. This could potentially add to the literature that informs dietary supportive care interventions for cancer patients.

## Methods

### Study design

This study is a secondary analysis of a prospective cohort study conducted between 2014 and 2017 at the National Cancer Centre Singapore. The original study was designed to evaluate the contribution of biomarkers to cancer-related fatigue [20]. The study was approved by the SingHealth Institutional Review Board (CIRB 2014/754/B). Informed consent was obtained from all study participants.

### Inclusion/exclusion criteria

The inclusion criteria for the original study included having a diagnosis of early-stage (I-III) breast cancer, being scheduled to receive standard chemotherapy, no prior history of chemotherapy or radiotherapy, and ability to understand English or Chinese. The exclusion criteria included having been diagnosed with metastatic cancer, being on medications that could lead to fatigue as a side effect (e.g., beta-blockers), or having other medical conditions that could cause fatigue (e.g., anemia), as the focus of the original study was fatigue. The current secondary analysis only selected participants (*n* = 31) that had available biomarker data on n-3 PUFAs.

### Data collection

Assessments utilized in this study took place before treatment initiation (baseline: T1) and at least 6 weeks after T1 during chemotherapy (T2). Patients completed a series of questionnaires at both timepoints, available both in English and Chinese, assessing health-related QoL. Demographic and medical information was collected through electronic databases and patient interviews. To quantify GI symptoms, we utilized the European Organisation for Research and Treatment of Cancer Quality of Life Questionnaire in Cancer Patients (EORTC QLQ-C30), which assesses GI symptoms (nausea and vomiting, appetite loss, diarrhea, and constipation) in the past week on a 4-point Likert-type scales (“not at all” to “very much”). Converted scores range from 0 to 100, with higher scores representing worsened symptoms or high level of symptomatology. Changes in symptom scores were calculated by subtracting T1 from T2 scores (T2–T1). The EORTC QLQ-C30 has been previously validated in breast cancer patients in Singapore [21, 22].

### Biomarker analyses

Blood samples (10 mL) were drawn to quantify levels of biomarkers; only T1 samples were utilized for the current analyses. Samples were kept in EDTA tubes, centrifuged at 1069 × *g* within 40 min of collection for a duration of 10 min, after which plasma was collected and then kept frozen at −80 degrees Celsius until analyses were performed. Aliquots of plasma were only thawed once before analysis.

- Levels of interleukin (*IL*)*-1b* (level of detection (LOD) 0.24 pg/mL), *IL-2* (LOD 0.75 pg/mL), *IL-3* (LOD 0.13 pg/mL), *IL-4* (LOD 0.09 pg/mL), *IL-6* (LOD 0.34 pg/mL), *IL-8* (LOD 0.36 pg/mL), *IL-10* (LOD 1.06 pg/mL), tumor necrosis factor-alpha (*TNF-α*) (LOD 1.13 pg/mL), interferon-gamma (*IFN-γ*) (LOD 1.05 pg/mL), and monocyte chemoattractant protein-1 (*MCP-1*) (LOD 0.44 pg/mL) were quantified using multiplex immunoassay (Luminex™) [23].
- Eicosapentaenoic acid (*EPA*) (LOD 2.42 ng/mL) [24], docosahexaenoic acid (*DHA*) (LOD 5.51 pg/mL) [25], and free fatty acid (*FFA*) (LOD 1 ng/mL) [26] levels were quantified from plasma using enzyme-linked immunosorbent assay (ELISA). Omega-3% (n-3%) was calculated ([DHA+EPA]/FFA). FFA levels were used to calculate n-3% and were not included in other analyses.

### Statistical analysis

Descriptive statistics were used to summarize patients’ demographic information, change of symptom scores over time, proportion of patients with GI symptoms, and characteristics of n-3 and inflammatory cytokines levels in this sample. Inflammatory markers with 10% or more missing data were excluded from analyses. Pearson’s correlation tests were used to assess the relationship between cytokines, n-3 PUFAs, and GI symptom changes. The Pearson correlation coefficient *r*, which ranges from −1 to 1, was used to determine the directionality (positive or negative) and strength (0.0–±0.3 weak; ±0.3–±0.7 moderate; ±0.7–±1 strong) of the correlation [27]. Paired sample *t-*tests were used to compare symptom mean scores across timepoints. Similarly, one-way analysis of variance (ANOVA) and post hoc Tukey’s HSD test were used to compare T1 DHA mean levels across patients whose symptoms had improved, remained the same, or worsened. Finally, multiple linear regressions were used to evaluate the predictive value of n-3 and pro-inflammatory markers on GI symptom changes (T2-T1 scores), adjusting for BMI and age, which have been found to affect the incidence and interference of GI symptoms in patients undergoing chemotherapy [28–30]. Analyses were conducted using IBM SPSS v28.0 and considered significant with two-sided *P-*values < 0.05.

## Results

### Demographics and baseline biomarkers

A total of 31 stage I-III breast cancer patients were included in the analysis. Majority of patients were Chinese (83.9%), married (64.5%), had secondary education (54.8%), spoke English (74.2%), were pre-menopausal (61.3%), had been diagnosed with stage II cancer (87.1%), and received anthracycline-based (89.6%) chemotherapy. Mean age ± standard deviation (SD) was 50.5 ± 8.8. Mean body max index (BMI) ± SD was 23.3 ± 3.8. BMI was classified as healthy (18.5 < 24.9), overweight (25 < 29.9), or obese (30+), with most participants (74.2%) within the healthy range (Table 1). Baseline plasma level of omega-3 (DHA, EPA, n-3%) and inflammatory biomarkers (IL-1b, IL-2, IL-4, IL-6, IL-8, IL-10, TNF-α, IFN-γ, and MCP-1) are described in Supplementary Table 1. Plasma inflammatory markers with 10% or more missing data were excluded from future analyses.

**Table 1:** Demographic characteristics

Variable or characteristic	*N*	Mean ± SD
Age	31	50.5 ± 8.8
BMI	31	23.3 ± 3.8
	***N***	Frequency (%)
BMI range		
Healthy	23	74.2
Overweight	6	19.3
Obese	2	6.5
Race		
Chinese	26	83.9
Malay	3	9.7
Indian	1	3.2
Other	1	3.2
Marital status		
Single	5	16.1
Married	20	64.5
Divorced	4	12.9
Widowed	2	6.5
Education		
None	1	3.2
Primary	3	9.7
Secondary	17	54.8
Pre-university	5	16.1
Graduate +	5	16.1
Language		
English	23	74.2
Chinese	8	25.8
Cancer stage		
Stage I	1	3.2
Stage II	27	87.1
Stage III	3	9.7
Menopause		
Pre-menopause	19	61.3
Post-menopause	12	38.7
Chemo regimen		
Anthracycline-based	25	89.6
Taxane-based	6	19.4

Abbreviations: *BMI* body mass index, *SD* standard deviation

### Prevalence of gastrointestinal (GI) symptom worsening

An overall increase in all GI symptom mean scores was observed between T1 and T2, indicating worsened symptoms (Table 2). Analysis showed statistically significant differences between T1 and T2 in nausea, vomiting, and diarrhea, but not in appetite loss or constipation. Additionally, appetite loss, nausea, and vomiting symptoms worsened in 32.3% of participants, while constipation and diarrhea symptoms worsened in 29% and 25.8% of participants, respectively. All participants whose scores worsened did so by 16.67% or more, which exceeded the clinical significance threshold of 10%.

**Table 2:** Trajectory of GI symptoms across timepoints

	Mean ± SD			Paired sample *t*-test
	T1	T2	T2–T1	Sig. 2-tailed
Appetite loss	13.98 (18.81)	24.73 (33.30)	+10.75 (35.89)	.106
Nausea and vomiting	4.30 (8.57)	13.44 (22.94)	*+9.14 (23.90)*	*.042*
Constipation	9.68 (15.38)	15.05 (20.80)	+5.38 (21.25)	.169
Diarrhea	1.08 (5.99)	10.75 (19.98)	*+9.68 (21.42)*	*.017*
	Improved	Remained the same		Worsened
Appetite loss	19.4%	48.4%		32.3%
Nausea and vomiting	12.9%	54.8%		32.3%
Constipation	12.9%	58.1%		29%
Diarrhea	3.2%	71%		25.8%

Abbreviations: *SD* standard deviation

Italicized values *p* < .05

### Correlation of symptoms and biomarkers

#### Omega-3 and inflammation

There were no statistically significant correlations observed between baseline n-3 PUFA biomarkers (DHA, EPA, n-3%) and pro-inflammatory cytokine levels (IL-8, TNF-α, MCP-1) (Table 3).

**Table 3:** Correlation between omega-3 biomarkers and inflammatory biomarkers

		*r*	
	DHA	EPA	n-3%
IL-8	−0.020	−0.093	−0.074
TNF-α	0.182	0.095	0.135
MCP-1	0.086	−0.049	0.021

*p < .05

**p < .01

#### GI symptom changes and biomarkers

Relationships between n-3 PUFA biomarkers and GI symptom changes (T2-T1), as well as between inflammatory biomarkers and GI symptom changes, are summarized in Table 4. A statistically significant strong positive correlation between DHA and change in appetite loss was found. No other statistically significant linear relationships were found between n-3 PUFA biomarkers and changes in GI symptoms. Additionally, statistically significant moderate positive correlations were found between IL-8 and change in appetite loss, IL-8 and change in nausea and vomiting, and MCP-1 and changes in appetite loss. In other words, higher IL-8 is correlated with greater appetite loss and greater nausea and vomiting, and higher MCP-1 is correlated with greater appetite loss. No additional statistically significant linear relationships between inflammatory markers and symptom changes were found.

**Table 4:** Correlation between GI symptom changes and biomarkers—omega-3 and inflammation

	*r*					
	n-3%	DHA	EPA	IL-8	MCP-1	TNF-α
T2-T1 appetite loss	.278	.512**	−.149	.478**	.384*	.309
T2-T1 nausea and vomiting	−.170	−.029	−.130	.438*	.242	.349
T2-T1 constipation	−.061	.124	−.025	.141	.116	.206
T2-T1 diarrhea	.097	−.034	.216	.147	.033	−.239

*p < .05.

**p <. 01

#### Comparison of DHA levels in patients with various GI trajectories

When we compared the mean T1 DHA levels across patients whose appetite loss [1] improved, [2] remained the same, or [3] worsened, our analysis showed a statistically significant difference in mean T1 DHA levels between the three groups (*p* = 0.024) (Table 5). Post hoc comparison indicated that mean T1 DHA levels for patients whose appetite loss improved (*M* = 29.43, *SD* = 23.66) was significantly lower than mean T1 DHA levels for patients whose appetite loss worsened (*M* = 226.94, *SD* = 175.44). There was no statistically significant difference between patients whose appetite loss improved and patients whose appetite loss remained the same (*M* = 156.76, *SD* = 119.68), nor between patients whose appetite loss remained the same and patients whose appetite loss worsened.

**Table 5:** Differences in DHA levels based on appetite loss changes

Comparison			
Control	Condition	Mean difference	Standard error
Improved	– Remained the same	−127.33	63.27
	– Worsened	−197.52*	67.64
Worsened	– Improved	197.52*	67.64
	– Remained the same	70.18	53.47

*p < .05

**p < .01

### Predictors of GI symptom change

Multiple linear regression modeling was used to evaluate if DHA and IL-8 at baseline significantly predicted changes in appetite loss from T1 to T2, correcting for age and BMI (Table 6). The overall regression was statistically significant (*R*^2^ = 0.565, *p* < .001), and it was found that DHA (standardized *β* = 0.586, *p* < .001) and IL-8 (standardized *β* = 0.497, *p* < .001) significantly predicted changes in appetite loss. Likewise, multiple linear regression was used to test if DHA and MCP-1 significantly predicted appetite loss changes, correcting for age and BMI. The overall regression was statistically significant (*R*^2^ = 0.425, *p* = .005), and DHA (standardized *β* = 0.540, *p* = .002) was found to significantly predict appetite loss changes. MCP-1 did not significantly predict changes in appetite loss. Finally, multiple linear regression was used to test if DHA and IL-8 at baseline significantly predicted changes in nausea and vomiting from T1 to T2, correcting for age and BMI. The overall regression was statistically significant (*R*^2^ = 0.368, *p* = .015), with IL-8 (standardized *β* =0.539, *p* = .002) significantly predicting nausea and vomiting changes. DHA did not significantly predict changes in nausea and vomiting, but BMI was found to be a statistically significant confounder (standardized *β* = 0.409, *p* = .017). Regression analyses for changes in constipation and changes in diarrhea were not performed, considering no statistically significant correlations were found between these symptoms and n-3 PUFA markers nor inflammatory markers.

**Table 6:** Predictors of gastrointestinal symptom change

	Δ Appetite loss *R*^2^ = 0.565, *p* < .001			Δ Appetite loss *R*^2^ = 0.425, *p* = .005			Δ Nausea and vomiting *R*^2^ = 0.368, *p* = .015		
	*β*_1_	*β*_2_	*p*	*β*_1_	*β*_2_	*p*	*β*_1_	*β*_2_	*p*
DHA	*0.586*	*.145*	*< .001*	*0.540*	*0.134*	*.002*	−0.018	−0.003	.911
MCP1	–	–	–	0.313	1.453	.050	–	–	–
IL8	*0.497*	*8.902*	*< .001*	–	–	–	*0.539*	*6.436*	*.002*
Age	0.203	0.832	.143	0.200	0.820	0.211	−0.182	-0.498	.270
BMI	0.163	1.527	.234	0.083	0.782	.585	*0.409*	*2.553*	*.017*

*β*_*1*_, standardized β. *β*_*2*_, unstandardized β

Italicized values *p* < .05

## Discussion

Our results discovered a statistically significant association between DHA, IL-8, MCP-1, and changes in appetite loss and between IL-8 and changes in nausea and vomiting, in early-stage breast cancer patients undergoing chemotherapy. All correlations were positive, indicating that larger DHA, IL-8, and MCP-1 baseline levels were associated with a larger increase in symptom scores (worsened symptoms) during chemotherapy. These relationships were supported by our regression results; DHA and IL-8, but not MCP-1, were found to significantly predict changes in appetite loss from baseline to 6 weeks after, during anti-cancer treatment. Similarly, IL-8 was found to significantly predict changes in nausea and vomiting, along with BMI. Again, with all positive regression coefficients, the direction of these relationships indicates that higher baseline levels of DHA and IL-8 predict worsened symptoms. More research examining the relationship between DHA and inflammation prior to chemotherapy and GI symptomatology progression is warranted to evaluate how interventions can target these biomarkers to improve GI symptoms in patients receiving chemotherapy.

Inflammatory processes have been studied as related to the formation of GI symptoms, both in cases related and unrelated to cancer and its treatment [31]. Considering this, the relationship that we have observed between baseline IL-8, a pro-inflammatory marker, and worsened symptoms is expected. Previous studies have shown significant associations between pro-inflammatory markers and GI toxicities in cancer patients, with evidence pointing to interactions between the immune system and the gut microbiome as responsible for symptom development [9, 13]. In agreement, Brown and colleagues conclude that the wide array of symptoms experienced by breast cancer patients, including neurocognitive and GI symptoms, along with pain and neuropathy, seem to be interconnected and mediated by inflammatory responses [13]. Likewise, in patients with geriatric cachexia, a complex metabolic syndrome characterized by excessive loss of body mass, pro-inflammatory markers such as TNF-α, IL-1, and IL-6 have been linked to symptoms such as decreased gastric motility and emptying, nausea, and vomiting [32]. Although these studies do not mention IL-8 specifically, it is known that cytokines rarely act alone; they stimulate cells that lead to secretion of other cytokines in a cascade-like manner and often have overlapping effects [33]. In that regard, our results agree with these studies.

Considering the role of inflammation in GI symptom formation, studies have explored anti-inflammatory interventions. Relatedly, it has been reported that n-3 PUFAs may lead to anti-inflammatory processes, with n-3 PUFA supplementation associated with improved appetite, weight, body composition, and nutritional status [19, 34–37]. Taking this literature into account, it was hypothesized that n-3 biomarkers, including DHA, would be inversely associated with inflammatory markers and, thus, inversely associated with symptom progression. However, according to our results, we did not observe any significant linear relationship between baseline DHA (nor other n-3 PUFA biomarkers) and baseline inflammatory markers. Additionally, although there was a statistically significant relationship between DHA and appetite loss, its directionality does not support our hypothesis: Our findings show that higher baseline DHA levels predict worsened appetite. Relatedly, patients whose appetite worsened had significantly higher mean DHA level than patients whose appetite improved, in disagreement with what was expected based on previous literature.

It is worth noting that though DHA was significantly associated with decreased appetite, no significant relationship was found for EPA nor n-3%, an index comprised of DHA+EPA. Most studies analyzing the effect of n-3 PUFAs in inflammation mention the potential benefits of both DHA and EPA [19]. However, there is growing evidence that these fatty acids differently regulate cytokine expression, with DHA more effectively lowering individual pro-inflammatory cytokines (TNF-α, IL-1β, IL-6, IL-18) and triglycerides and EPA playing a more significant role in balancing the pro-inflammatory to anti-inflammatory cytokine ratio [38–41]. However, the directionality of the relationship between appetite loss and DHA remains puzzling. Limitations in our study, detailed in the following paragraph, provide possible explanations for these results.

Our findings are limited to the demographic characteristics of this sample: female, Asian, early-stage breast cancer patients, and mostly receiving anthracycline-based chemotherapy. Our results should be interpreted with caution considering the following limitations. First, our findings are limited by a small sample size. Relatedly, additional possible confounders such as chemotherapy regimen, cancer stage, and menopause could be considered in a larger sample. This would allow for a more accurate evaluation of the relationship between baseline n-3 PUFAs and inflammatory markers in symptom progression. Second, with DHA and EPA obtained from plasma rather than from whole blood, there may be variations in levels captured. It is also important to note that the measured EPA values fell below the range of detection, making these values and those derived from EPA (n-3%) less reliable. Likewise, n-3 PUFA levels in cell membranes were not considered. Third, in relation to inflammatory biomarkers, our data confined to the analyses of MCP-1, IL-8, and TNF-α. Having more available data points for a wider sample of pro-inflammatory cytokine levels would allow for more rigorous conclusions. Considering all the above, more studies are needed to further clarify the relationship between baseline n-3 levels, baseline pro-inflammatory cytokine levels, and GI symptom trajectories in patients undergoing chemotherapy.

Though our findings need to be interpreted in the context of the listed limitations, to our knowledge, no previous studies have analyzed baseline inflammatory markers and n-3 levels and their relationship with GI symptom progression in breast cancer patients undergoing anticancer treatment. Considering the prevalence of cancer- and treatment-related toxicities and their detrimental impact on treatment outcomes and sense of well-being, gaining a better understanding of the baseline factors that play a role in symptom trajectories is important. Findings in this matter can be used to inform interventions that, even before beginning chemotherapy, could prevent or diminish the development of chemotherapy-associated GI symptomatology and, thus, improve treatment success and QoL.

## Conclusion

We observed that baseline levels of IL-8 and DHA predicted gastrointestinal symptom trajectories (such as appetite loss and nausea and vomiting) in breast cancer patients undergoing chemotherapy. Higher levels of inflammatory markers predicted worsened appetite loss and nausea and vomiting, and higher levels of DHA predicted worsened appetite loss. More studies are needed to better understand the predictive value of these biomarkers in chemotherapy-related gastrointestinal symptom progression, as well as to test the therapeutic potential of interventions targeting these biomarkers to prevent the onset of chemotherapy-related gastrointestinal symptoms.

### Supplementary information

ESM 1 (PDF 210 kb)

## Data Availability

The data that support the findings of this study are available from the corresponding author upon reasonable request.
