# Supplementary material for: Prediction of gastrointestinal symptoms trajectories using omega-3 and inflammatory biomarkers in early-stage breast cancer patients receiving chemotherapy
Source: Support Care Cancer. 2024 Jan 3;32(1):76. doi: 10.1007/s00520-023-08274-5 (PMC10764400; doi:10.1007/s00520-023-08274-5)
Supplement: Supplementary file 1 — (PDF 210 kb) [file 520_2023_8274_MOESM1_ESM.pdf]

Supplementary Table 1. Omega-3 and Inflammatory Markers Levels at Baseline					
	**N**	**Minimum**	**Maximum**	**Mean**	**Standard Deviation**
**DHA (pg/mL)**	31	10.50	531.50	154.75	144.54
**EPA (pg/mL)**	31	123.00	516.10	273.44	106.22
**FFA (ng/mL)**	31	5.86	30.85	12.06	5.46
**n-3 (%)**	31	1.01	9.55	3.99	1.99
**IL-1b (pg/mL)**	19	0.01	1.05	0.33	0.33
**IL-2 (pg/mL)**	9	0.02	2.16	0.72	0.70
**IL-4 (pg/mL)**	16	0.01	0.82	0.28	0.26
**IL-6 (pg/mL)**	24	0.02	3.18	0.93	0.93
**IL-8 (pg/mL)**	31	0.15	7.64	3.03	2.00
**IL-10 (pg/mL)**	4	0.20	9.43	2.68	4.51
**IFN-γ (pg/mL)**	24	0.01	39.07	11.20	13.45
**TNF-α (pg/mL)**	30	0.05	23.17	7.74	6.97
**MCP-1 (pg/mL)**	31	2.35	32.58	19.37	7.74
